# The Conceptual Model and Methods of Wave 1 (2016) of the EUREST-PLUS ITC 6 European Countries Survey

**DOI:** 10.18332/tid/99881

**Published:** 2018-12-12

**Authors:** Geoffrey T. Fong, Mary E. Thompson, Christian Boudreau, Nicolas Bécuwe, Pete Driezen, Thomas K. Agar, Anne C. K. Quah, Witold A. Zatoński, Krzysztof Przewoźniak, Ute Mons, Tibor Demjén, Yannis Tountas, Antigona C. Trofor, Esteve Fernández, Ann McNeill, Marc Willemsen, Constantine I. Vardavas, on behalf of the EUREST-PLUS consortium

**Affiliations:** 1 University of Waterloo, Waterloo, Canada; 2 Ontario Institute for Cancer Research, Toronto, Canada; 3 Kantar Public (TNS), Brussels, Belgium; 4 Health Promotion Foundation, Warsaw, Poland; 5 European Observatory of Health Inequalities, President Stanisław Wojciechowski State University of Applied Sciences, Kalisz, Poland; 6 Maria Sklodowska-Curie Institute - Oncology Center, Warsaw, Poland; 7 Cancer Prevention Unit and WHO Collaborating Centre for Tobacco Control, German Cancer Research Center (DKFZ), Heidelberg, Germany; 8 Smoking or Health Hungarian Foundation (SHHF), Budapest, Hungary; 9 National and Kapodistrian University of Athens (UoA), Athens, Greece; 10 University of Medicine and Pharmacy ‘Grigore T. Popa’ Iasi, Iasi, Romania; 11 Aer Pur Romania, Bucharest, Romania; 12 Tobacco Control Unit, Catalan Institute of Oncology (ICO) and Cancer Control and Prevention Group, Bellvitge Biomedical Research Institute (IDIBELL), L’Hospitalet de Llobregat, Catalonia, Spain; 13 School of Medicine and Health Sciences, University of Barcelona, Catalonia, Spain; 14 Addictions Department, King’s College London, London, United Kingdom; 15 UK Centre for Tobacco and Alcohol Studies, King’s College London, London, United Kingdom; 16 Maastricht University, Maastricht, the Netherlands; 17 European Network for Smoking and Tobacco Prevention (ENSP), Brussels, Belgium; 18 University of Crete (UoC), Heraklion, Greece

**Keywords:** tobacco control, evaluation, cohort study, EU Tobacco Products Directive, ITC Project

## Abstract

Population-level interventions represent the only real approach for combatting the tobacco epidemic. There is thus great importance in conducting rigorous evaluation studies of tobacco control policies and regulations such as those arising from the WHO Framework Convention on Tobacco Control (FCTC) and the European Union’s 2014 Tobacco Products Directive (TPD). The ITC 6 European Countries Survey, a component of the Horizon 2020 Project entitled *European Regulatory Science on Tobacco: Policy Implementation to Reduce Lung Disease (EUREST-PLUS)*, was created to evaluate and impact of the TPD in six EU Member States: Germany, Greece, Hungary, Poland, Romania, and Spain. In each country, a cohort survey of a representative national sample of 1000 smokers was conducted. This paper describes the conceptual model, methodology, and initial survey statistics of Wave 1 of the ITC 6E Survey, which was conducted June–September 2016. The ITC 6E Survey’s conceptual model, methodology, and survey instrument, were based on the broader 29-country ITC Project cohort studies, which have been conducted since 2002. The commonality of methods and measures allow a strong potential for cross-country comparisons between the 6 EU countries of the ITC 6E Project and 3 other EU countries (England, France, The Netherlands) in the ITC Project, as well as the broader set of ITC countries outside the EU.

## INTRODUCTION

Given the enormity of the global tobacco epidemic, the most effective approach for reducing its devastation is to create and implement strong interventions at the level of entire populations. The WHO Framework Convention on Tobacco Control (FCTC) is the most prominent of these efforts, given its global reach (with 180 countries and the European Union as FCTC Parties) and the comprehensive set of measures contained within the treaty text, which have been developed to a greater extent through the adoption of guidelines for a number of policy domains including: price/tax, smoke-free, labeling and packaging, advertising, promotion, sponsorship, cessation, and curbing illicit trade. In 2016, an independent expert group concluded its impact assessment of the WHO FCTC in the first decade of the treaty. They concluded that the WHO FCTC has led to significant increases in the implementation of tobacco control measures, and that when implemented, key demand-reduction measures of the treaty had led to a significant decline in smoking prevalence^1-3^.

The second most extensive population-level effort in tobacco control is the European Union’s (EU) 2014 Tobacco Products Directive (TPD) (2014/40/EU). The 2014 EU TPD updated existing regulations from the previous EU TPD of 2001 (2001/EC/37), under which all EU Member States (MS) had to meet minimum standards on a number of tobacco control regulatory issues including, but not limited to, tobacco labelling, product design and packaging restrictions, ingredient and emission reporting for tobacco, roll-your-own, smokeless tobacco products, e-cigarettes, novel tobacco products, waterpipe tobacco and herbal smoking products with an implementation date of 20 May 2016, with some regulations allowing for implementation as late as May 2017.

In order to assess the impact of these two large tobacco control efforts at a population level, the EUREST-PLUS Project *European Regulatory Science on Tobacco: Policy Implementation to Reduce Lung Disease*, aims to monitor and evaluate the updated 2014 EU TPD, and its associated implementing acts, within the context of WHO FCTC ratification at a European level. The main objective of the EURESTPLUS Project is to monitor and evaluate the impact of the implementing acts of the EU TPD and assess these within the context of WHO FCTC ratification at a European level. A key aspect of the project is the ITC 6 European Country (6E) Surveys—a cohort survey evaluating the psychosocial and behavioural impact of the implementation of the EU TPD Articles and policies outlined in the WHO FCTC among a cohort of smokers in six EU MS^4^.

The present work describes the conceptual framework and methods used by the ITC 6 European Country (6E) Survey to evaluate the regulations of the EU TPD.

## METHODS

Evaluation of tobacco control policies and regulations Population-level interventions such as policies and regulations cannot be evaluated through randomized experiments. However, there are important methodological and design strategies that can be used to increase the internal validity of non-experimental/observational studies to evaluate the impact of policies, these are: 1) a cohort design in which individuals are measured on the same key outcome variables over time, critically before and after the introduction of the policy (a pre-post design); 2) a quasi-experimental design (i.e. ‘natural experiments’, or in the language of economists, a ‘difference-in-difference design’), in which one group exposed to a policy is compared to a group that has not been exposed, i.e. a pre-post design with control groups (such as other countries)^5,6^; 3) the measurement of appropriate policy-specific variables (‘proximal variables’) that are conceptually close to the policy being evaluated and less likely to be affected by other factors.

The International Tobacco Control Policy Evaluation (ITC) Project, created in 2002, is an international evidence system that has evaluated WHO FCTC policies across 29 countries, covering over half of the world’s population and over two-thirds of the world’s tobacco users. The ITC Project was the first, and is still the only, international research program with a focus on WHO FCTC impact evaluation.

The ITC Project incorporates all three strategies in its research design. These three innovative strategies, with the inclusion of other explanatory variables (covariates), include design and content features that are unparalleled in the study of population-level interventions and produce a research design with the potential to make strong inferences about policy impact^7^. Evaluation studies conducted by the ITC Project have provided a large body of evidence on the effectiveness of WHO FCTC policies including price/tax policies^8,9^, graphic health warnings^10-12^, elimination of ‘light/mild’ brand descriptors^13^, comprehensive smoke-free laws^14-18^, advertising/promotion bans^19^, impact of plain packaging on effectiveness of health warnings^20^, and cessation policies^21^.

### Conceptual framework of the ITC Surveys

**Figure 1:**
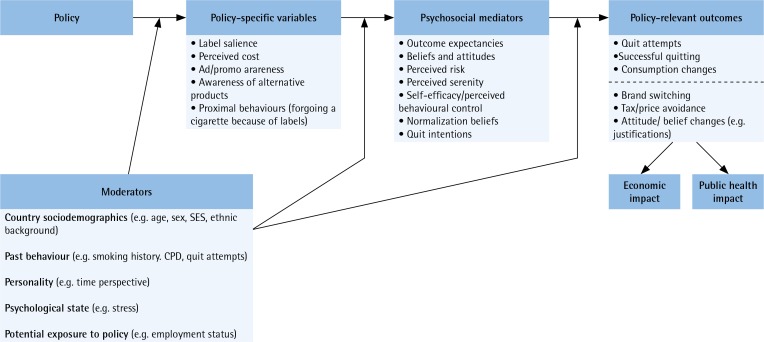
The ITC Conceptual Model used in the construction of the ITC Surveys^22^

The ITC Project is founded on a strong theory-driven conceptual framework that provides the context for the survey content, hypotheses, and data analysis^7,22-24^. The ITC Conceptual Model is presented in Figure 1:

1. *Policies* are seen as affecting a variety of psychosocial and behavioral variables. The most immediate effects are those on the *policy-specific variables* (proximal variables), the variables most connected with the policy itself. Changing from text-only warnings to larger graphic warnings should increase the salience of warnings, as well as other variables that are among the objectives of warnings, e.g. more frequent thoughts about the harms of smoking and about quitting; price should affect the perceived cost of cigarettes (e.g. belief that cigarettes have become too expensive). These effects on the policy-specific variables are the first step in the causal chain. In addition to the *convergent validity* that multiple measures of impact of a given policy provide, their *specificity* to a single policy offers the important tool of *discriminant validity* for distinguishing impact of a single policy in the context of other active policies. For example, the measure ‘warnings make me think about the health risks of smoking’ was specifically designed to measure changes in health warnings, and not for smoke-free laws or price increases. This specificity allows for greater internal validity in evaluating impact of multiple policies^7^.
2. *Psychosocial mediators* (distal variables) are the next step in the causal chain. They are known to predict smoking behavior and quitting, and come from well-known psychosocial health behavior models, e.g. Theory of Planned Behavior^25^, Social Cognitive Theory^26^, the Health Belief Model^27^, and Protection Motivation Theory^28^. Policies affect these variables indirectly via effects on policy-specific variables.
3. *Outcome variables* include beneficial outcomes (e.g. quitting) but also responses that may not lead to the intended public health benefits, e.g. after a tax increase, smokers may switch to cheaper brands, which confers no public health benefit.

The ITC Conceptual Model is thus a causal chain model. In the domain of warnings, if switching to graphic warnings leads to increased quitting^29^, this occurs by first increasing the warning-specific variables (e.g. extent to which warnings make the smoker think about risk). Those changes in the warning-specific variables then lead to changes in psychosocial mediators (e.g. quit intentions). The third step is from those mediators to behavioral outcomes such as quit attempts. Thus, policy affects behavior because it causes changes in the policy specific variables, which in turn cause changes in the psychosocial mediators. Thus, we seek to understand how and why policies have impact, not just whether they have impact.

The cohort design allows us some ability to test the causal chain of effects depicted in the model through mediational analysis; whereas a repeat cross-sectional design does not^7,22^. ITC researchers have used structural equation modeling to validate the mediational model in various ITC countries for health warnings^30^ and smoke-free laws^18^.

*The ITC Six European Country (6E) Survey* is a multi-wave European-focused cohort study with the purpose of measuring the direct and indirect impacts of the EU TPD, and policies of the WHO FCTC. The six countries participating in the ITC 6E Survey are Germany, Greece, Hungary, Poland, Romania, and Spain.

We now describe the methods of data collection and recruitment, the sample sizes and sampling designs, sampling protocols, constructions of survey weights, and survey outcome rates. More detailed information on these topics is available in the ITC 6E Survey Technical Report^31^.

### Sample design and features

The ITC 6E Survey was designed to produce nationally representative samples in each of the six project countries, with a sample size of 1000 smokers from each country in Wave 1. Respondents would qualify if they were of age 18 years or older, smoked at least monthly, and smoked over 100 cigarettes in their lifetime. All interviews were conducted face-to-face using a computer (tablet)-assisted personal interview (CAPI) mode. Survey respondents were interviewed between 18 June and 16 September 2016. In each country, the target number of respondents was either met or exceeded by a few (Table 1).

**Table 1:** Total number of interviewed respondents by country

*Country*	*Sample size*
Germany	1003
Greece	1000
Hungary	1000
Poland	1006
Romania	1001
Spain	1001
Overall	6011

### Sample selection

The probability sample in each country was chosen by dividing each country into major geographic regions, i.e. Nomenclature of Territorial Units for Statistics or *Nomenclature des unités territoriales statistiques*, which is a geocode standard for referencing subdivisions of EU countries (NUTS2 regions; NUTS1 in Germany). The Greek Islands in the Ionian Sea, The Northern Aegean Sea, and Southern Aegean Sea were excluded from the sample in Greece, as were the Canary Islands, Ceuta, and Melilla from Spain. The geographic strata were made up of the NUTS regions crossed with the degree of urbanization, that is whether the region was urban, semi urban-rural, or rural. The strata were conceptually conceived to be a grouping of clusters, each the size of an enumeration area. The numbers of clusters to be sampled were allocated to strata approximately proportionally to the size of the population of age 18 years or older. The objective in the design was to sample 100 random clusters within each country, with at least two clusters in each stratum. Within each cluster, 10 adult smokers would be interviewed.

In each cluster, interviewers used a random walk method to select each dwelling to be approached. The tablet program selected a starting point at random using only GPS coordinates, and a random walk path. Following the designated path, interviewers approached every 5th address. In cases where the address selected contained multiple households, a random unit was selected. Four attempts to make contact were required for each selected dwelling unit before a new address could be selected.

When a household was contacted, a knowledgeable informant was identified to provide the number of male and female smokers aged 18 or older. Using the next birthday method^32^, one male smoker and one female smoker were selected for interview where possible. Should only one smoker of a given gender be available, that person was automatically selected. To ensure the randomness of the sample, no substitutions were allowed within a household, with the exception of replacement of selected individuals who were unavailable for the entirety of the fieldwork period. This process of screening, selection and interviewing continued within a cluster until the required number of smokers were interviewed.

### Survey development

As with all ITC surveys, the ITC 6E Wave 1 Survey was designed with the following aims in mind: 1) examine the impact of tobacco control policies, including the EU TPD and the WHO FCTC; 2) compare smoking behaviours and policy impact among the six countries and the wider group of ITC countries; and 3) gather evidence to suggest changes to current tobacco policy. To accomplish these aims, the ITC 6E Survey drew questions from several active ITC surveys that measure smoking-related behaviours, perceptions and attitudes toward quitting, policy-based measurements including questions on warnings, advertisements/promotion of tobacco products, and more. Due to the expanding scope of tobacco policy and related novel products, questions were added covering products such as electronic cigarettes and vaping devices, as well as heated tobacco devices. Finally, moderators and psychosocial measures were included that provide behavioural context, as well as basic demographic information.

The majority of the survey questions for the 6E Survey had already been used in ITC surveys conducted in other countries; this commonality was helpful to allow for valid cross-country comparisons. There were, however, a number of specific policy issues in the EU TPD that required new questions to be developed. Several teleconferences were held between EUREST-PLUS Project members to discuss, propose, and edit possibilities for appropriate survey questions to measure the impact of EU TPD regulations, as well as to make changes across the survey to be more appropriate for the European context and also across the diversity of the six countries (e.g. to make changes in terminology or phrasing because of differences across countries in how concepts are expressed).

Because of the objective of conducting pre-post evaluation analyses of the EU TPD, it was important that Wave 1 was conducted as early as possible to allow for data collection before the EU TPD was implemented, and that Wave 2 would be conducted after key measures and regulations of the EU TPD had been implemented (Wave 2 was conducted starting in late February 2018), to measure change over time. While the EU TPD compliance date in the declaration was 20 May 2016, some of the provisions of the EU TPD were allowed another year for implementation. The fieldwork of the Wave 1 survey began on 18 June. This meant that some of the EU TPD’s impact may have been experienced at the time of the Wave 1 survey. However, some of the provisions of the EU TPD had extended dates for full implementation, so the slight delay in the launching of Wave 1 was not very problematic. Wave 1 stands as an accurate benchmark of the time of implementation, and a strong point of comparison for future waves to measure the potential impact of the EU TPD.

### Survey management

The survey fieldwork for the ITC 6E Survey was managed by Kantar Public in Brussels, one of the partner organizations within the EUREST-PLUS Project. Kantar Public put out a public call for local survey teams within each of the six countries, as required by the EUREST-PLUS funding mechanism, the Horizon 2020 Funding Scheme. The fieldwork was conducted in the 6 European countries by the following agencies: Foerster and Thelen (Germany), Metron Analysis (Greece), Kantar TNS Hoffman (Hungary), Kantar TNS Polska (Poland), Curs (Romania), and Kantar TNS Spain (Spain).

Kantar Public oversaw the translation of all survey materials. With final scripts completed in English, the translation into the six national languages was handled in-house. For each language, the questionnaire was translated first by an independent translator, at which point the survey went through a two-stage revision period: first, via a second independent proof-reader; and second, by the national agency project manager. Upon completion, the translated survey was sent to the respective country team members of the ITC 6E Country Project to ensure accuracy.

Kantar Public was also responsible for overseeing the training of the local fieldwork agencies listed above. Representatives from Kantar trained local team members on the use of Kantar’s Computer Assisted Personal Interview (CAPI) survey software, ensuring that fieldwork operators could follow the fieldwork procedures, both in sampling and in interviewing respondents.

The protocol at each approached household followed seven basic steps: A basic introduction; the administration of the household screener to a knowledgeable individual – a short survey to ensure that qualified respondents resided within the household; an information and consent procedure for selected respondents; the administration of an individual screener to the selected respondents to ensure qualification; the administration of the full ITC 6E Survey; and a thank you including remuneration for the respondents’ time. Incentive amounts were established in consultation with local survey teams and based on best practices for each country (10€ in Germany, Hungary and Poland, 7€ in Romania, 5€ in Greece, and 3€ in Spain), see Table 2.

**Table 2:** Respondent remuneration by country

*Country*	*Incentive (€)*
Germany	10
Greece	5
Hungary	10
Poland	10
Romania	7
Spain	3

Due to the international nature of the project, fieldwork timeframes varied from country to country both in start and completion dates (Table 3). Fieldwork in Greece was suspended for one week (12 August to 19 August) to accommodate the summer vacation period within the country, as a significant amount of the population was unavailable during this time, it was important to postpone fieldwork to ensure accurate representativeness.

**Table 3:** Fieldwork start and end dates (all in 2018)

*Country*	*Start date*	*End date*
Germany	15 June	30 August
Greece	16 June	12 September
Hungary	22 June	20 July
Poland	25 June	21 August

The survey protocols and all materials, including the survey questionnaires, were cleared for ethics by the Office of Research Ethics, University of Waterloo, Canada and by local ethics boards in the participating countries. Written consents from respondents were obtained.

### Quality control

Monitoring the fieldwork to ensure quality control was achieved through multiple checks. First, fieldwork in all countries was managed centrally by Kantar Public in Brussels. Field data were transmitted via NFIELD software to Kantar, allowing them to monitor and assess progress in real time. Second, Fieldwork Progress Reports were provided by the Project Manager to Kantar Public, who then sent weekly updates to the University of Waterloo, Canada, for additional monitoring. Third, Field Supervisors were appointed and charged with supervising interviewers and assisting them with any questions or issues related to fieldwork protocol. Finally, at the local level, 10% back checks were conducted on interviews conducted within a week of completion. Here, checks on coding and comparisons between raw samples of data and national populations were conducted ensuring item response was 100% within the completed surveys.

### Data protection

The database of the survey responses are identified only by a unique ID number, without participants’ identifiable information. All data analyses were conducted on the de-identified data.

### External data sharing

Data from this project are available to approved researchers starting two years after the date of issuance of cleaned data sets by the ITC Data Management Centre at the University of Waterloo. Researchers interested in using ITC 6E data are required to apply for approval by submitting an International Tobacco Control Data Repository (ITCDR) request application and subsequently to sign an ITCDR Data Usage Agreement.

### Survey weights

It is standard among ITC datasets to construct weights that correct and adjust for sample misrepresentation due to factors such as unequal sampling probabilities, frame error, and non-responses. It is also desirable to improve the precision of the datasets estimates through the application of information available via auxiliary sources, for instance sociodemographic benchmarks^33^.

In order to compute sampling weights for each smoker participating in the survey, first, each respondent was assigned an initial weight equal to the reciprocal of their probability of being selected within their household. Second, the probability of the respondent’s inclusion within the stratum was estimated. As the design for sampling dwellings was a random walk procedure, the probability of inclusion of a dwelling or household is proportional to the number of random walks in which the household would be sampled. Since these numbers were unknown, the inclusion probability of a household was taken to be approximately equal within each stratum, and thus the inclusion probability of an individual within the stratum was taken to be proportional to the selection probability within the household. Third, a post-stratification adjustment based on estimated smoker prevalences from Eurobarometer 2014 was performed to calibrate weights among stratum, sex, and age groups. Finally, for analytic use, particularly those involving comparisons across the six countries, as well as with other ITC country datasets, the weights were rescaled to have a mean equal to 1 in each country. To account for the complexity of the sampling design in the estimation of standard errors and computation of confidence intervals, bootstrap weights were provided based on the Rao and Wu (1998)^34^ technique.

### Survey outcome rates

Table 4 shows the calculation of household and individual response rates and cooperation rates. Household contact rates were high, except in Romania; the household response rates were moderate, being somewhat lower in Germany and Greece, where there were evidently greater proportions of refusals to have eligibility determined. Household and individual cooperation rates were high.

**Table 4:** Survey outcome rates

	*Germany*	*Greece*	*Hungary*	*Poland*	*Romania*	*Spain*
1. Number of addresses approached/attempted	10325	3537	2754	3421	4490	4114
2. Number of addresses where contact was made	8259	2856	2404	3028	2778	3853
3. Number of contacted addresses with eligibility determined	3086	1295	1791	2154	2065	2451
4. Number of contacted addresses with no eligible respondents	1982	466	972	1163	1134	1509
5. Number of contacted addresses with eligible respondents	1104	829	819	991	931	942
6. Number of addresses with eligible respondents, members selected	1085	823	809	976	911	936
7. Eligibility rate for households, given determination of eligibility (5./3.)	0.358	0.640	0.457	0.460	0.451	0.384
8. Estimated eligible households among attempted (7.*1.)	3694	2264	1259	1574	2024	1581
9. Number of individuals selected for interview	1425	1116	1078	1265	1254	1234
10. Number of individual refusals or break offs	200	91	64	227	232	219
11. Number of completed interviews	1003	1000	1000	1006	1003	1001
12. Household contact rate (2./1.)	0.800	0.807	0.873	0.885	0.619	0.937
13. Household cooperation rate, given eligible (6./5.)	0.983	0.993	0.988	0.985	0.979	0.994
14. Household response rate (6./8.)	0.294	0.363	0.642	0.620	0.450	0.592
15. Individual cooperation rate (11./(11.+10.))	0.834	0.917	0.940	0.816	0.812	0.820
16. Individual response rate, given selection (11./9.)	0.704	0.896	0.928	0.795	0.800	0.811

## DISCUSSION

Wave 1 of the ITC 6 European Country Survey employed the same conceptual model, as well as identical or functionally similar methods that have been employed across all 29 countries of the ITC Project. This consistency across countries over time allows for strong comparability of the findings from these six EU countries to the three other EU countries participating in the ITC Project (United Kingdom, Netherlands, France) and across the 20 non-European ITC countries. The data from Wave 2, conducted beginning in February 2018, will allow for important pre-post evaluation studies of the EU TPD and other tobacco control measures.

## CONCLUSIONS

The ITC 6 European Country Survey, and the broader EUREST-PLUS Project, holds great potential to provide important evidence regarding the impact of measures designed to reduce tobacco use, a major cause of premature death and disease in Europe and throughout the world.

## CONFLICTS OF INTEREST

The authors declare that they have no competing interests, financial or otherwise, related to the current work. N. Bécuwe reports grants from Kantar Public Brussels, during the conduct of the study. K. Przewoźniak reports grants and personal fees from the Polish League Against Cancer, outside the submitted work. C. I. Vardavas reports that he is the Strategic Development Editor of TID and that there are no conflicts of interest with this current work. The rest of the authors have also completed and submitted an ICMJE form for disclosure of potential conflicts of interest.
